# Hydrocele of the Canal of Nuck with Endometriosis: Right-Side Dominance Confirmed by Literature Review and Statistical Analysis

**DOI:** 10.1155/2020/2567267

**Published:** 2020-05-04

**Authors:** Shunsuke Nagase, Kanako Ogura, Karin Ashizawa, Asumi Sakaguchi, Ryo Wada, Toshiharu Matsumoto

**Affiliations:** ^1^Department of Diagnostic Pathology, Juntendo University Nerima Hospital, 3-1-10 Takanodai, Nerima, Tokyo, Japan; ^2^Department of Pathology, Juntendo University Shizuoka Hospital, 1129 Nagaoka, Izunokuni, Shizuoka, Japan

## Abstract

*Introduction*. The canal of Nuck is an embryological remnant of the peritoneal pouch that extends into the labium majus of women. Hydrocele is the most common presentation, but only a small number of cases are reported in association with endometriosis. *Case Presentation*. The patient is a 45-year-old woman who presented with left inguinal mass with persistent pain. Computed tomography (CT) and magnetic resonance imaging (MRI) revealed a 30 mm cystic mass, and a hydrocele of the canal of Nuck (HCN) was suspected. The excised mass was a cyst containing yellow-tan serous fluid, and the cyst wall was lined by mesothelium. The morphology was consistent with conventional HCN. However, since several foci of endometrial-like epithelium and stroma were identified beneath the mesothelium, the mass was diagnosed with HCN with endometriosis (EM-HCN). *Discussion*. Right-side dominance of EM-HCN is suggested by several authors, but a thorough review has never been performed. For the first time, we reviewed the literature and statistically confirmed that EM-HCNs dominantly occur on the right side compared to those without endometriosis. We consider that this supports the theory that endometriosis derives from retrograde menstruation of endometrial tissue through fallopian tubes. When endometriosis is discovered in HCN, the clinician should be aware of the possibility of pelvic endometriosis.

## 1. Introduction

Embryologically, in female, the parietal peritoneum extends into the inguinal canal and the labium majus accompanying the round ligament of the uterus; however, it usually obliterates completely within the first year of life. When it fails to obliterate, the structure is called the canal of Nuck, named after a Dutch anatomist Anton Nuck who described the first case in 1691 [1]. The canal of Nuck is an uncommon anomaly which is analogous to patent processus vaginalis in male, and it can cause a hydrocele or an indirect inguinal hernia depending on the diameter of the canal. Hydrocele of the canal of Nuck (HCN) is occasionally confused with inguinal hernia as they both typically present as a painless groin mass. However, unlike inguinal hernia, HCN does not contain bowel or fat; Doppler ultrasonography is considered useful for the diagnosis [2].

Endometriosis is a common gynecologic disorder accounting for 6 to 10% of the general female population, and it is characterized by the presence of endometrial-like epithelium and stroma outside the endometrium. The peritoneum and pelvic organs are well-known sites of endometriosis being the cause of chronic pelvic pain, dysmenorrhea, and infertility, yet distant locations such as pleura can also be affected potentially triggering catamenial pneumothorax [3].

Rarely, endometriosis is discovered in HCN. Several authors have suggested that HCN with endometriosis (EM-HCN) tends to occur more on the right side and presents with pain compared to those without endometriosis (non-EM-HCN) [4–8]. However, a thorough review has never been performed. Here, we report a case of EM-HCN with a review of the literature and aim to prove the right-side dominance by statistical analysis for the first time.

## 2. Case Presentation

**Figure 1:**
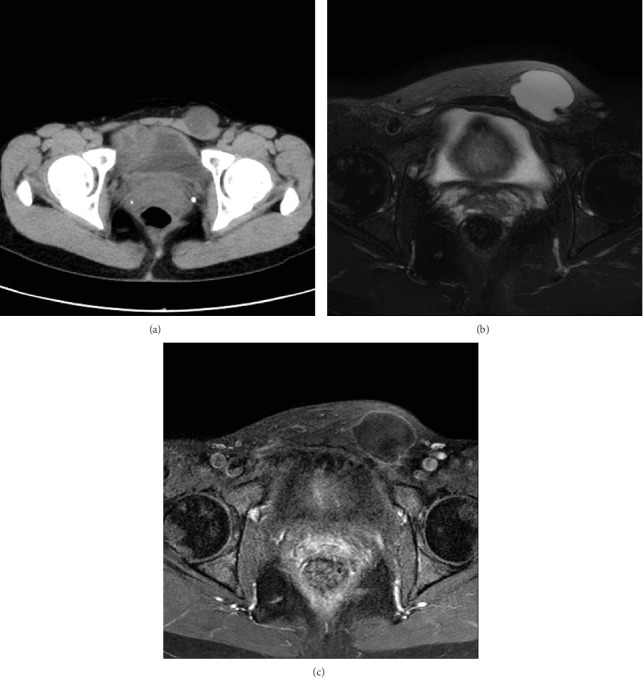
(a) CT displaying a 30 mm low-density mass in the left inguinal region. (b) Fat-suppressed T2-weighted MRI displaying a subcutaneous mass with homogeneous high-intensity signal. (c) Gadolinium-enhanced fat-suppressed T1-weighted MRI revealing a well-circumscribed cystic mass with a thin wall.

The patient is a 45-year-old woman without any particular medical history. She noticed a left inguinal mass a week before and visited our hospital because of persistent pain. The patient did not recall any episode of trauma. On examination, the mass was firm, smooth, and irreducible. Computed tomography (CT) and magnetic resonance imaging (MRI) displayed a 30 mm cystic mass in the subcutis of the left inguinal region which does not communicate with the peritoneum (Figures 1(a)–1(c)). Hydrocele of the canal of Nuck was suspected. The mass was excised and submitted for a pathological study.

**Figure 2:**
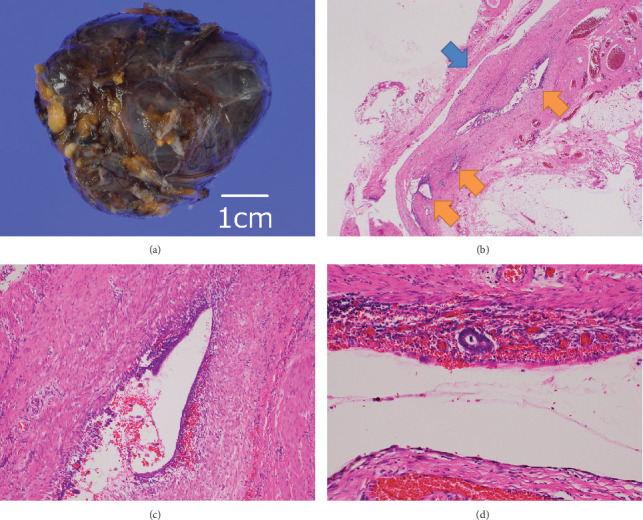
(a) Macroscopic image of the mass containing yellow-tan serous fluid. (b) Several foci of endometriosis (orange arrows) beside the cyst (blue arrow). (c) Endometrial-like epithelium and stroma accompanying hemorrhage and hemosiderin-laden macrophages. (d) Endometriosis beneath single-layered mesothelium in high-power field.

Macroscopically, it was a cyst with a dark brown surface containing yellow-tan serous fluid (Figure 2(a)). The cyst wall was smooth and thin, and no solid component was identified. Microscopically, the cyst was lined by single-layered flat cells which were considered as mesothelium; it was a typical morphology of HCN. However, a few glands of columnar cells accompanied by round monotonous cells that were considered as endometrial tissue were also identified beneath the mesothelium in several areas as well as hemorrhage and hemosiderin-laden macrophages (Figures 2(b)–2(d)).

**Figure 3:**
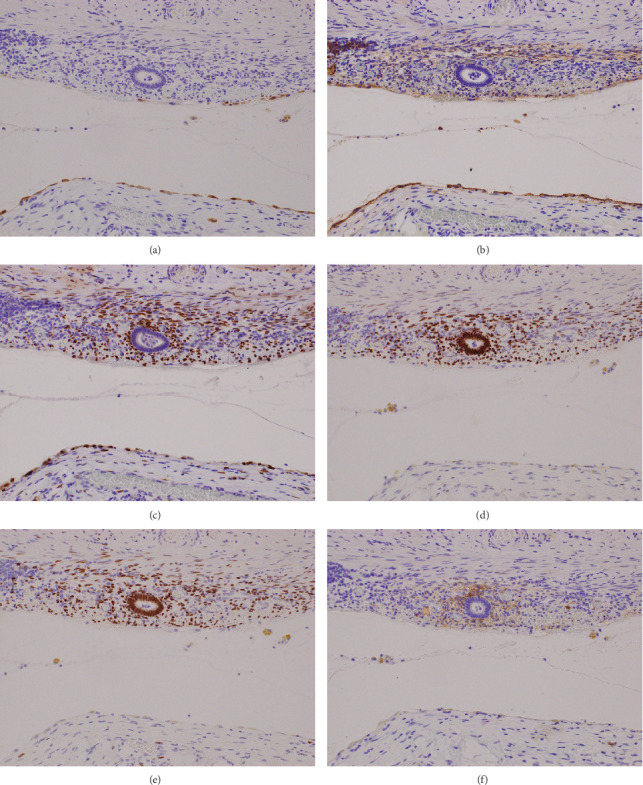
(a) Calretinin; (b) podoplanin; (c) WT-1; (d) ER; (e) PR; (f) CD10.

In immunohistochemistry, single-layered flat cells were positive for calretinin, podoplanin, and Wilms' tumor 1 (WT-1) (Figures 3(a)–3(c)). Columnar cells were positive for estrogen receptor (ER) and progesterone receptor (PR) (Figures 3(d) and 3(e)). Round monotonous cells were positive for ER, PR, CD10, and WT-1 (Figures 3(d)–3(f) and 3(a)). These results were consistent with mesothelium and endometrial tissue, respectively.

Based on the histology and the result of immunohistochemistry, the mass was diagnosed with EM-HCN. The postoperative course was uneventful, and the patient was discharged two days after the operation. No sign of recurrence was observed in the follow-up after three weeks.

## 3. Review of the Literature and Statistical Analysis

We reviewed English-reported cases of both EM-HCN and non-EM-HCN that were published in 2000 or later [6–32]. Only cases of reproductive age (age: 15-49) reported with definite pathological diagnosis were included. Cases complicated with infection or neoplasm were excluded. The reviewed cases are summarized in Table 1.

Out of 25 cases of EM-HCN including our case (age: 20-46, mean: 34.6), 21 cases (84%) occurred on the right side. In contrast, out of 28 cases of non-EM-HCN (age: 17-45, mean: 34.0), only 16 cases (57%) occurred on the right side (Table 2). Using Fisher's exact test, we discovered that EM-HCN dominantly occurred on the right side compared to non-EM-HCN with a significant difference (84% vs. 57%, *p* = 0.041). The analysis was performed with EZR (Saitama Medical Centre, Jichi Medical University, Saitama, Japan) [33].

## 4. Discussion

Right-side dominance of the inguinal endometriosis has been suggested by several authors reporting that 86-90% occurred on the right side [4, 5]. Nevertheless, statistical analysis has never been attempted.

So far, two theories are proposed as the pathogenesis of endometriosis: one is the metaplasia of coelomic epithelium and the other is the retrograde menstruation of endometrial tissue through fallopian tubes [3]. The former theory cannot logically explain the dominance of the right-sided EM-HCN; however, based on the latter theory, Candiani et al. introduced an idea that the clockwise flow of the ascites makes the right side more susceptible to endometrial implants and left side protected by the sigmoid colon [5]. This idea is adopted from catamenial pneumothorax in which 87.5 to 100% of the cases are reported on the right side [34].

Regarding the pain, either discomfort or pain was reported in 12 cases (48%) of EM-HCN and 7 cases (25%) of non-EM-HCN. EM-HCNs tend to present with pain more as it has been suggested in the past. However, we could not analyze these cases as the status of pain was not reported in 15 cases (33%).

Among EM-HCNs, catamenial symptom such as pain and change of size were present in 7 cases (28%), absent in 16 cases (64%), and not reported in 2 cases (8%). Interestingly, catamenial symptom was not reported in any of the non-EM-HCN indicating that the presence of catamenial symptom may help a clinician suspect the complication of endometriosis.

Also, MRI is considered useful in detecting endometriosis in the ovary and HCN typically showing high signal intensity on T1-weighted image and shading sign on T2-weighted image [7]. Nevertheless, in our case, MRI showed non-EM-HCN pattern, reflecting the nature of the fluid, and could not detect endometriosis preoperatively. The sensitivity of MRI is also discussed by some authors highlighting the significance of pathological examination [8, 28].

In addition, out of 25 cases of EM-HCN, pelvic endometriosis was discovered in 10 cases (40%). Thus, it is important to inform the clinician the possibility of pelvic endometriosis when EM-HCN is revealed pathologically. This is especially important because patients with HCN are frequently referred to a general surgeon and not a gynecologist after initially being diagnosed with hernia.

## 5. Conclusion

We reported a rare case of EM-HCN with a review of the literature and statistically confirmed that EM-HCNs dominantly occur on the right side compared to non-EM-HCNs for the first time. This supports the theory that endometriosis derives from retrograde menstruation of endometrial tissue through fallopian tubes.

Endometriosis in HCN can be identified pathologically even if it is absent in MRI. When endometriosis is discovered in HCN, the clinician should be aware of the possibility of pelvic endometriosis.

## Figures and Tables

**Table 1:** Literature review of EM-HCN and non-EM-HCN.

Endometriosis	Author	Year	Age	Side	Pain/discomfort	Pelvic endometriosis	Catamenial symptom
(-) (non-EM-HCN)	Yen et al.	2001	33	R	(-)	NR	NR
	Yen et al.	2001	28	R	(-)	NR	NR
	Yen et al.	2001	23	R	(-)	NR	NR
	Khanna et al.	2007	17	R	(+)	NR	NR
	Ozel et al.	2009	38	R	NR	NR	NR
	Caviezel et al.	2009	28	R	(-)	NR	NR
	Jagdale et al.	2012	35	R	(+)	NR	NR
	Bunni et al.	2013	38	R	(+)	NR	NR
	Kono et al.	2015	43	R	(-)	NR	NR
	Heer et al.	2015	31	R	(-)	NR	NR
	Patnum et al.	2016	38	R	(-)	NR	NR
	Ferreira et al.	2017	23	R	(-)	NR	NR
	Heng et al.	2018	31	R	NR	NR	NR
	Aalberg et al.	2018	44	R	NR	NR	NR
	Aalberg et al.	2018	46	R	(+)	NR	NR
	Chihara et al.	2019	38	R	(-)	NR	NR
	Yen et al.	2001	45	L	(-)	NR	NR
	Yen et al.	2001	37	L	(-)	NR	NR
	Yen et al.	2001	26	L	(-)	NR	NR
	Wei et al.	2002	26	L	(+)	NR	NR
	Bhattacharjee et al.	2006	24	L	(-)	NR	NR
	Qureshi et al.	2014	28	L	(+)	NR	NR
	Pandey et al.	2014	42	L	(-)	NR	NR
	Matsumoto et al.	2014	37	L	(-)	NR	NR
	Topal et al.	2018	42	L	NR	NR	NR
	Aalberg et al.	2018	40	L	NR	NR	NR
	Aalberg et al.	2018	37	L	NR	NR	NR
	Lucas et al.	2019	33	L	(+)	NR	NR

(+) (EM-HCN)	Cervini et al.	2005	31	R	(+)	(-)	(+)
	Kirkpatrick et al.	2006	41	R	NR	NR	NR
	Bagul et al.	2011	43	R	(+)	(-)	(-)
	Jimenez et al.	2011	35	R	(+)	(-)	(+)
	Okoshi et al.	2017	44	R	(+)	(+)	(-)
	Niitsu et al.	2019	38	R	NR	(-)	(-)
	Niitsu et al.	2019	25	R	NR	(-)	(-)
	Niitsu et al.	2019	31	R	NR	(-)	(-)
	Niitsu et al.	2019	40	R	(+)	(-)	(+)
	Niitsu et al.	2019	44	R	NR	(-)	(-)
	Niitsu et al.	2019	36	R	(+)	(-)	(+)
	Niitsu et al.	2019	20	R	NR	(-)	(-)
	Niitsu et al.	2019	38	R	NR	(-)	(-)
	Gaeta et al.	2010	22-46 (mean 30)	R : L = 8 : 0	(+): (‐) = 4 : 4	(+): (‐) = 8 : 0	(+): (‐) = 2 : 6
	Wang et al.	2009	35	L	(+)	(+)	(+)
	Niitsu et al.	2019	43	L	NR	(-)	(-)
	Niitsu et al.	2019	35	L	NR	(-)	(-)
	Current case	2020	45	L	(+)	(-)	NR

R: right; L: left; NR: not reported.

**Table 2:** Laterality of the reviewed cases.

	Right	Left	Total
EM-HCN	21	4	25
Non-EM-HCN	16	12	28
Total	37	16	53
